# MiR‐379‐5p inhibits the proliferation, migration, and invasion of breast cancer by targeting KIF4A

**DOI:** 10.1111/1759-7714.14437

**Published:** 2022-05-24

**Authors:** Ke Yang, Danyang Li, Weihui Jia, Yanmei Song, Ningxin Sun, Jiemin Wang, Hongli Li, Chonggao Yin

**Affiliations:** ^1^ College of Nursing Weifang Medical University Weifang China; ^2^ Medicine Research Center Weifang Medical University Weifang China

**Keywords:** breast cancer, invasion, KIF4A, miR‐379‐5p, proliferation

## Abstract

**Background:**

Many studies have shown that microRNAs (miRNAs) play an essential role in gene regulation and tumor development. This study aimed to explore the expression of miR‐379‐5p and its mechanisms of affecting proliferation, migration, and invasion in breast cancer (BC).

**Methods:**

MiRNAs and mRNAs expression data of BC and normal breast tissue samples were downloaded from the TCGA and GEO databases. qRT‐PCR was used to detect the expression of miR‐379‐5p in human normal breast epithelial cell lines and human BC cell lines. The proliferation ability of transfected cells was detected by colony formation and EdU assays. The mobility and invasion ability of transfected cells was measured by wound healing and transwell assays. The relative protein expression of transfected cells was detected by western blot. Dual luciferase reporter assay was performed to identify the targeted binding of miR‐379‐5p and KIF4A.

**Results:**

MiR‐379‐5p was lowly expressed in BC tissue samples and BC cell lines. The target genes of miR‐379‐5p were involved in many cancer‐related signaling pathways. PPI analysis and the cytoHubba algorithm of Cytoscape identified 10 genes as the hub genes. Survival analysis showed that only KIF4A expression in 10 hub genes was significantly associated with the prognosis of BC patients and was significantly upregulated in BC. Overexpression of miR‐379‐5p inhibited proliferation, migration, and invasion in the BC cell line MDA‐MB‐231, which could be reversed by KIF4A.

**Conclusions:**

MiR‐379‐5p inhibits proliferation, migration, and invasion of BC by targeting KIF4A.

## Graphical abstract

MiR‐379‐5p is inversely correlated with the expression of KIF4A. MiR‐379‐5p inhibits the proliferation, migration and invasion of breast cancer by targeting KIF4A.

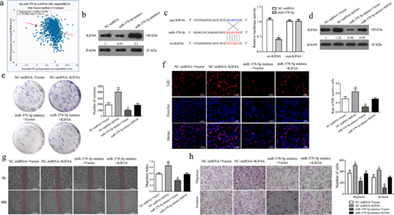

## INTRODUCTION

Breast cancer (BC) is the most common cancer among women in many countries and one of the leading causes of cancer‐related deaths in women, with more than one‐tenth of new cancers diagnosed each year being BC.^1^ Although significant progress has been made in preventing, diagnosing, and treating BC in recent years, current evidence‐based medicine shows that BC treatment and prognosis are still not ideal due to the high recurrence rate and poor prognosis.^2^, ^3^ Therefore, it is crucial to understand the molecular pathogenesis of BC and find new therapeutic targets and biomarkers.^4^

MicroRNAs (miRNAs) are short RNA molecules 19 to 25 nucleotides in size that regulate post‐transcriptional silencing of target genes. The single miRNA can target hundreds of mRNA and affect the expression of many genes, which are usually involved in the functional interaction pathway.^5^ At present, more and more miRNAs are abnormally expressed in various types of cancer cell lines and clinical tumor specimens.^6^ Abnormally expressed miRNAs may be involved in the pathological development of specific tumor phenotypes by regulating the expression of particular genes.^7^ Scientists have been developing robust and safe targeting approaches to restore these suppressive miRNAs in cancer cells.^8^ The recovery of downregulated miRNAs or missing in cancer is called miRNA replacement therapy.^9^ The restored miRNAs can inhibit the expression of mRNAs, which encodes critical oncoproteins.^10^ Therefore, the further study of miRNAs is helpful for the prediction, diagnosis, and prognosis of cancer in the future.^11^ MiR‐379‐5p is differentially expressed in various cancer tissues and cells, such as hepatocellular carcinoma, prostate cancer, gastric cancer, lung cancer, and osteosarcoma.^12^, ^13^, ^14^, ^15^, ^16^ Studies have shown that the expression level of miR‐379 was significantly decreased in BC tissues compared with normal breast tissues and significantly negatively correlated with cyclin B1.^17^

KIF4A, a kinesin superfamily member (KIFs), belongs to the KIFA subfamily.^18^ KIF4A plays an essential role in chromosome positioning, spindle organization, DNA damage repair, and other functions.^19^, ^20^, ^21^ However, it is not clear whether miR‐379‐5p regulates the biological activity of BC cells by regulating KIF4A.

This study explored BC miRNA expression profiles by array data from GEO and TCGA databases and explored miRNA‐mRNAs interactions in BC. We examined the biological effects of increased miR‐379‐5p on BC and predicted KIF4A that binds to miR‐379‐5p. MiR‐379‐5p exhibited its cancer suppressive function by targeting KIF4A in BC cells. Overexpression of KIF4A could reverse the inhibitory effects of miR‐379‐5p induced BC cell proliferation, migration, and invasion. In summary, our results demonstrate that miR‐379‐5p and KIF4A may serve as promising therapeutic targets for BC.

## METHODS

### Microarray data

The expression of microarray datasets GSE61438, GSE48088, GSE59247, and GSE65194 were obtained from the GEO database (https://www.ncbi.nlm.nih.gov/geo/). GSE61438 includes 44 BC tissues and 13 normal breast tissues. GSE48088 includes 33 BC tissues and 3 normal breast tissues. GSE59247 includes 44 BC tissues and 4 normal breast tissues. The GSE65194 of mRNA gene chip includes 130 BC tissues and 11 normal breast tissues. Other miRNAs and mRNA sequencing datasets were downloaded from TCGA (https://portal.gdc.cancer.gov/). The 1207 samples of miRNAs from TCGA included 1103 BC tissues and 104 normal tissue samples, and 1222 samples of mRNAs included 1109 BC tissues and 113 normal tissue samples.

GEO2R was applied to identify the differentially expressed miRNAs and mRNAs in the GEO database between BC and normal breast tissues. The adjusted *p* < 0.05 and |log2 fold change (FC)| ≥ 1 were set as the cutoff criteria of miRNAs microarray data. The adjusted *p* < 0.05 and |log2 FC| ≥ 2.5 were selected as the cutoff criteria of mRNAs microarray data. R software was used to analyze miR‐379‐5p and KIF4A expression.

### Online public database of expression and prognosis

StarBase (http://starbase.sysu.edu.cn/) and miRWalk (http://mirwalk.umm.uni-heidelberg.de/) databases were used to predict the target genes of miRNAs. The online drawing software (http://bioinformatics.psb.ugent.be/webtools/Venn/) was used to take the differentially expressed miRNAs and target genes. STRING online software (https://string-db.org/) was used to construct the protein–protein interaction (PPI) network. The PPI network was constructed after importing the TSV format data file into Cytoscape software to visualize the mRNA‐mRNA regulatory networks. The top 10 target genes of protein degree were selected as hub genes for the following analysis. Survival analysis of hub genes in BC was performed by UALCAN database (http://ualcan.path.uab.edu/index.html). The protein expression of hub genes in the sample tissue was obtained from the Human Protein Atlas (HPA) database (https://www.proteinatlas.org/). StarBase was used to analyze the correlation between miRNA and mRNA.

### Functional enrichment analysis of target genes

DAVID database (https://david.ncifcrf.gov/) was used for gene ontology (GO) enrichment analysis of the target genes. Kyoto Encyclopedia of Genes and Genomes (KEGG) pathway enrichment analysis of target genes was performed using the KOBAS database (http://kobas.cbi.pku.edu.cn/). GSEA software version 4.1.0 was used for gene set enrichment analysis (GSEA) to assess the significant pathways associated with the expression of KIF4A.^22^ Significantly enriched gene sets need to meet the following conditions: false discovery rates (FDR) < 0.25, *p* < 0.05.

### Cell culture and reagents

MCF‐10A, MCF‐7, MDA‐MB‐231, and HEK‐293 T cells were purchased from the American Type Culture Collection (ATCC, Manassas, VA, USA). MCF‐10A cells were cultured in mammary epithelial growth medium (Catalog no. CC‐3150). MCF‐7 and HEK‐293 T cells were cultured in Dulbecco's Modified Eagle Medium (Solarbio, Catalog no. 11995) and 1% sodium pyruvate. MDA‐MB‐231 cells were cultured in Roswell Park Memorial Institute 1640 medium (RPMI‐1640) (Solarbio, Catalog no. 3180). The four kinds of cells were cultured with 10% fetal bovine serum (FBS) (HyClone, SH30070.03) in a humidified atmosphere of 5% CO_2_ at 37°C.

### Transfection of BC cells

According to the manufacturer's protocol, corresponding negative control (NC‐miRNA), miR‐379‐5p mimics, miR‐379‐5p inhibitor, KIF4A, and Vector were purchased from GeneChem Company. According to the manufacturer's instructions, lipofectamine 2000 reagent (Invitrogen; Thermo Fisher Scientific) was used for transfection. Stable cells were cultured for further tests.

### RNA extraction and quantitative real‐time PCR (qRT‐PCR)

RNA extraction and qRT‐PCR were performed as previously described.^23^ U6 was used as an internal reference for qRT‐PCR. MiRNA relative expression was normalized as in the previous study.^24^ Sequences of primers were as follows, miR‐379‐5p RT primer: 5'‐GTCGTATCCAGTGCAGGGTCCGAGGTATTCGCACTGGATACGACCCTACG‐3'; miR‐379‐5p forward primer: 5'‐GCGCGTGGTAGACTATGGAA‐3', miR‐379‐5p reverse primer: 5'‐AGTGCAGGGTCCGAGGTATT‐3'; U6 forward primer: 5'‐CTCGCTTCGGCAGCACA‐3', U6 reverse primer: 5'‐AACGCTTCACGAATTTGCGT‐3'.

### 5‐Ethynyl‐2'‐deoxyuridine (EdU) assay

According to the manufacturer's instructions, the EdU assay (RiboBio) was carried out. Then, 24 h after transfection, each group's cells were added with the same volume of 10 mmol/l EdU as the medium and removed after 2 h of incubation. After cells were fixed with 4% paraformaldehyde for 15 min, they were incubated with 0.3% Triton X‐100 for 15 min at room temperature. Click reaction solution was added to each well and set in the dark for 30 min. Hoechst 33343 was used for nuclear staining followed by observation with a fluorescence microscope.

### Colony formation assay

Single‐cell suspension was obtained by trypsinization, and seeded in a 6‐well plate with a density of 1000 cells in each well. The cells were cultured in a 5% carbon dioxide incubator for 2 weeks, and the medium was kept fresh. After colony formation, the cells were fixed with 4% paraformaldehyde for 20 min and stained with 10% Giemsa.

### Wound healing assay

Wound healing assays were used to analyze the migration of transfected cells. MDA‐MB‐231 cells were seeded on a 6‐well plate. The damaged monolayers were washed with phosphate‐buffered solution to remove cell debris. After wound formation, the morphological changes of the cells in each well were observed at 0 and 48 h under the microscope. All assays were performed independently in triplicate.

### Transwell assay

A transwell assay was used to analyze the migration and invasion capabilities of cells. In this experiment, the cells were plated in the upper chamber with serum‐free medium. Then, 500 μl medium containing 10% FBS was added to the lower chamber as the chemoattractant. After being incubated for 24 h, the migrated cells were fixed with 4% paraformaldehyde and stained with Giemsa. For the invasion test, before plating the cells, we used the reconstituted basement membrane matrigel (BD) as the substrate for the invasion to add to the upper chamber. The remaining steps are similar to those previously described. The number of invading cells was calculated using the light microscope (Olympus). The experiments were performed in triplicate.

### Western blot

Total proteins were extracted from BC cell lines as previously stated.^25^ The protein samples were separated by sodium dodecyl sulfate‐polyacrylamide gel electrophoresis and transferred to polyvinylidene difluoride membranes (PVDF) (Millipore Inc). The PVDF membranes were blocked with 5% nonfat milk. Finally, the PVDF membranes were incubated with the specific primary antibody overnight at 4°C and incubated with the secondary antibody at 37°C for 1 h. The following antibodies were used in this study: β‐actin (1:5000), KIF4A (1:500). β‐actin was used for normalization. Antibodies were purchased from Abcam (www.abcam.cn). ImageJ software was used to evaluate and quantify the gray value of western blot.

### Luciferase reporter assay

The wild‐type or mutant 3'UTR of KIF4A was amplified and cloned separately into pmirGLO vector. The HEK‐293 T cells were cotransfected with wild‐type or mutant luciferase plasmids and miR‐379‐5p plasmid or NC‐miRNA. A dual‐luciferase reporter assay system (Promega) was used to measure the luciferase activity. All experiments were independently performed three times.

### Statistical analysis

Differences among groups were assessed by paired, two‐tailed Student's *t*‐test. *p* < 0.05 was considered statistically significant, and all statistical analyses were conducted using SPSS 26.0 software.

## RESULTS

### MiR‐379‐5p was aberrantly downregulated in BC tissues

**FIGURE 1:**
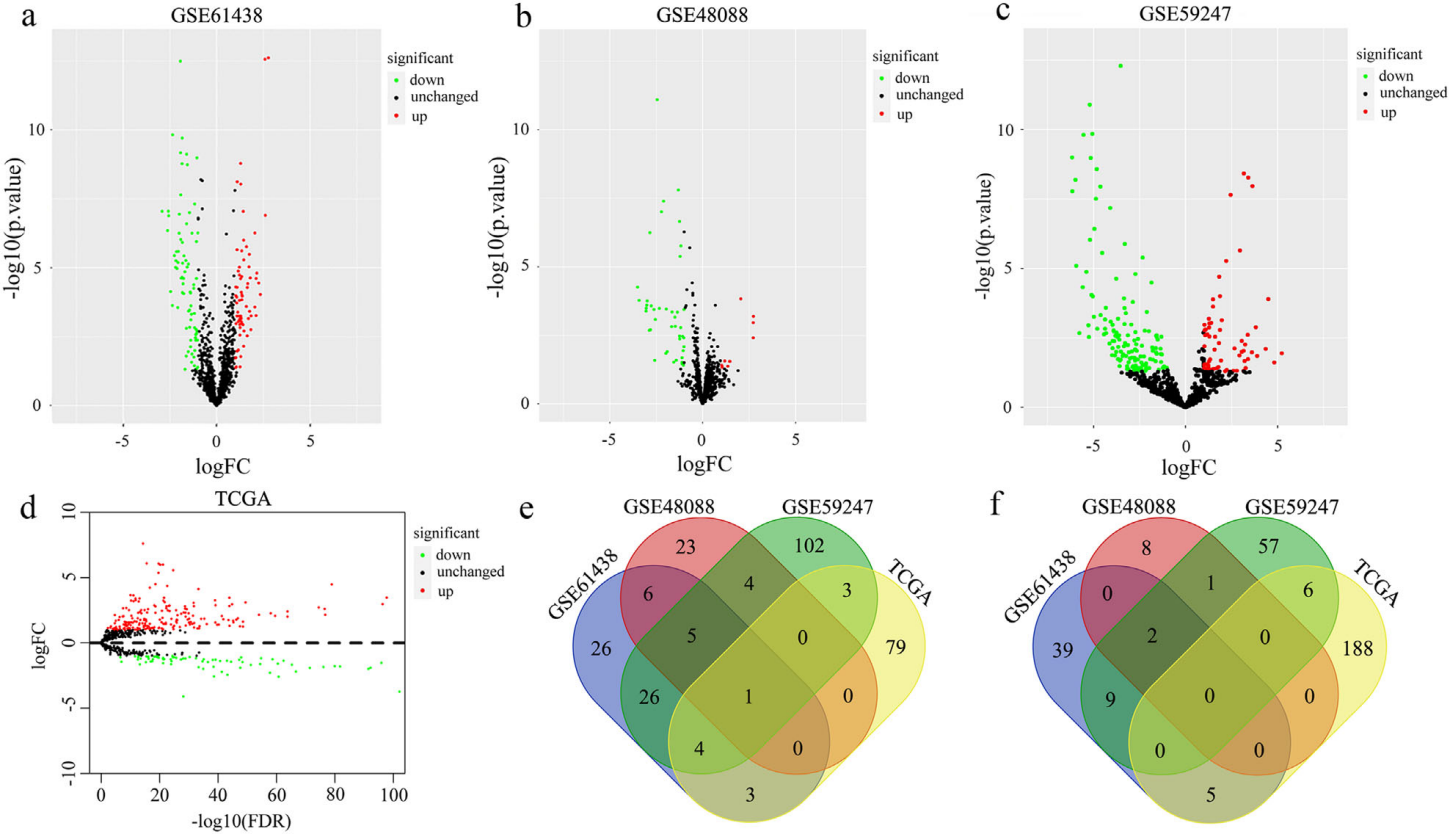
MiR‐379‐5p was aberrantly downregulated in BC tissues. (a) Volcano plot of differential expression miRNA analyses in GSE61438, (b) GSE48088, (c) GSE59247, and (d) TCGA. (e) Venn diagram of downregulated miRNAs in microarray datasets GSE61438, GSE48088, GSE59247, and TCGA. (f) Venn diagram of upregulated miRNAs in microarray datasets GSE61438, GSE48088, GSE59247, and TCGA

We analyzed differentially expressed miRNAs in microarray datasets GSE61438, GSE48088, and GSE59247. Differential expression analysis of GSE61438 showed 71 downregulated miRNAs and 55 upregulated miRNAs in BC tissues (Figure 1a). GSE48088 showed 39 downregulated miRNAs and 11 upregulated miRNAs in BC tissues (Figure 1b). GSE59247 showed 145 downregulated miRNAs and 75 upregulated miRNAs in BC tissues (Figure 1c). There were 90 downregulated miRNAs and 199 upregulated miRNAs in TCGA (Figure 1d). After taking the intersection, only one downregulated miRNA was found, namely miR‐379‐5p (Figure 1e). Moreover, no upregulated miRNAs were obtained (Figure 1f). Therefore, miR‐379‐5p was selected for the next exploration.

### Bioinformatic analyses of target genes for miR‐379‐5p

**FIGURE 2:**
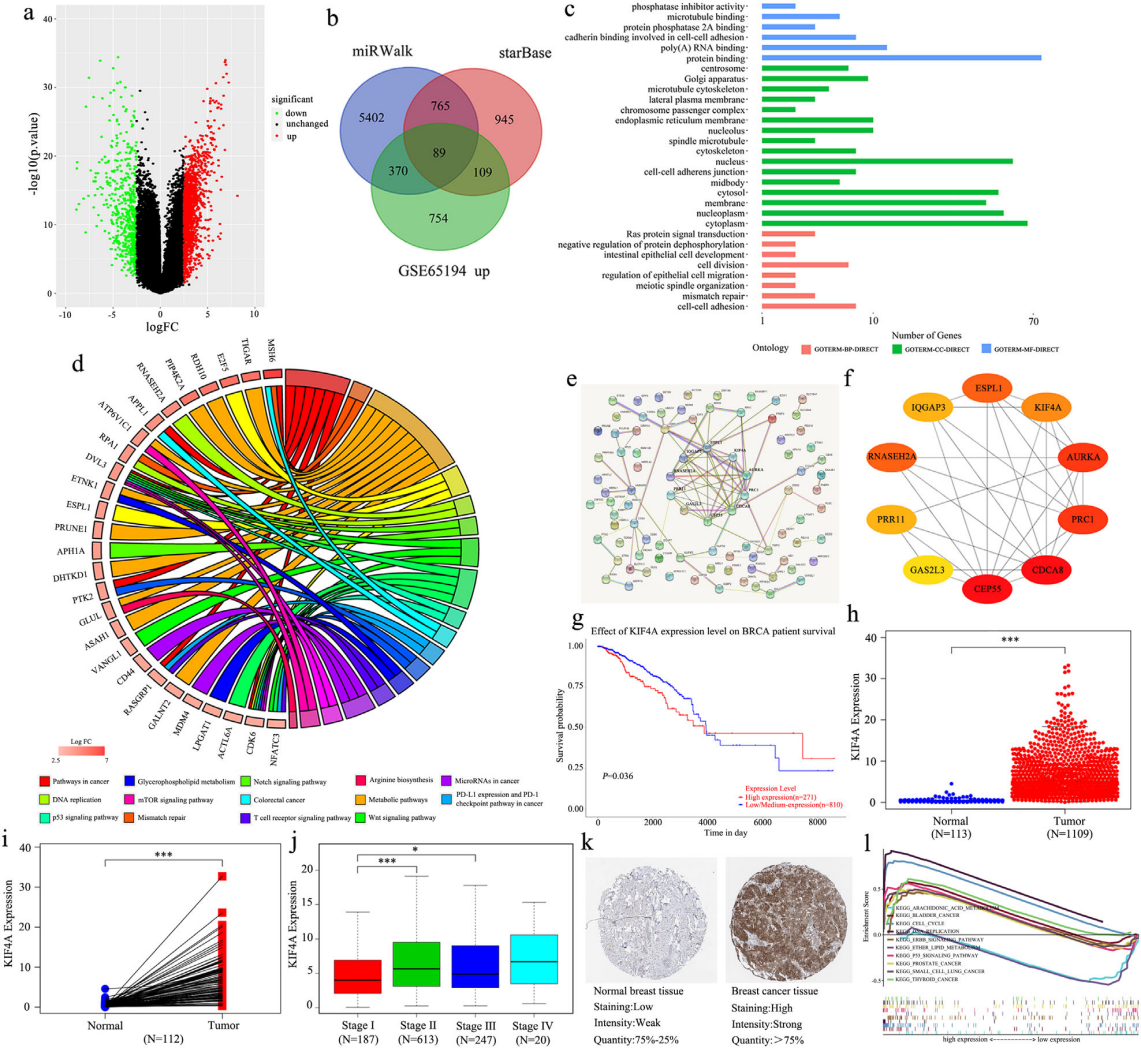
Bioinformatic analyses of target genes for miR‐379‐5p. (a) Volcano plot of differentially expressed mRNAs in microarray dataset GSE65194. (b) Venn diagram of upregulated genes in microarray data GSE65194 with target genes of miR‐379‐5p. (c) GO enrichment analysis of 89 target genes. (d) KEGG pathway enrichment analysis of 89 target genes. (e) The PPI network of 89 target genes. (f) Hub genes obtained by cytoHubba algorithm of Cytoscape. (g) The survival analysis is based on the expression of KIF4A in BC patients. (h) The expression levels of KIF4A in normal breast and BC tissue of TCGA. (i) The expression levels of KIF4A in cancer tissues and adjacent normal tissues from the same patients of TCGA. (j) The expression of KIF4A protein at different stages of BC tissues in TCGA (all data are presented as mean ± SD. *p < 0.05; *** *p* < 0.001). (k) The protein expression of KIF4A in the HPA database. (l) Enrichment plots of GSEA in BC with the high and low KIF4A expression phenotype

The microarray dataset GSE65194 containing mRNAs expression profiles was used to reference target gene expression levels. Differential expression analysis of GSE65194 showed that 1322 mRNAs were upregulated and 558 mRNAs were downregulated in BC (Figure 2a). The target genes of miR‐379‐5p were predicted by starBase and miRWalk databases, resulting in 89 target genes after crossing with 1322 upregulated mRNAs in GSE65194 microarray data (Figure 2b). These 89 target genes are essential to miR‐379‐5p and may regulate many mechanisms. To understand these target genes more fully, we conducted GO and KEGG pathway enrichment analysis and observed significant enrichment of various functions. GO enrichment analysis of target genes was divided into molecular function, cellular component, and biological process (Figure 2c). The molecular functions of target genes mainly include protein binding, poly RNA binding, etc. The cellular component shows that target genes were primarily enriched in the cytoplasm, nucleus, nucleoplasm, etc. The biological processes of target genes mainly include cell–cell adhesion, cell division, mismatch repair, etc. KEGG pathway enrichment analysis shows that target genes were primarily enriched in metabolic pathways, pathways in cancer, Wnt signaling pathway, p53 signaling pathway, PD‐L1 expressing and PD‐1 checkpoint pathway in cancer, and T cell receptor signaling pathway, etc (Figure 2d).

The STRING database is the online software commonly used to identify interactions between proteins. Analyzing 89 target genes in BC using the PPI network, PPI showed that 96 edges and 90 nodes were involved, and the average local clustering coefficient was 0.361 (Figure 2e). The interaction of these proteins was closely related and may have an important relationship with BC prognosis. Cytohubba is the plug‐in of Cytoscape software for calculating the degree of each protein node. The top 10 protein degrees were hub genes, namely ESPL1, KIF4A, IQGAP3, RNASEH2A, AURKA, PRR11, PRC1, GAS2L3, CDCA8, and CEP55 (Figure 2f). We analyzed the relationship between the expression of 10 hub genes and survival probability in patients. The results showed that BC patients with high KIF4A expression had a lower survival rate than those with low KIF4A expression (Figure 2g). Meanwhile, the expression of ESPL1, IQGAP3, RNASEH2A, AURKA, PRR11, PRC1, GAS2L3, CDCA8, and CEP55 was not significantly associated with the adverse prognosis of BC patients (Figure S1). Therefore, we mainly focused on KIF4A for further exploration. In the TCGA database, the expression of KIF4A in BC tissues was higher than that in normal breast tissues (Figure 2h). In addition, the expression level in cancer tissues from the same patients was also significantly higher than that in adjacent normal tissues (Figure 2i). Compared with stage I, stages II and III expression levels were significantly increased (Figure 2j). Next, the HPA database showed that the protein expression of KIF4A was upregulated in BC tissues compared to normal ones (Figure 2k). Single‐gene GSEA analysis confirmed that high expression of KIF4A was linked to the ErbB signal pathway, p53 signal pathway, cell cycle, DNA replication, etc. Low expression of KIF4A was linked to the ether lipid metabolism and arachidonic acid metabolism (Figure 2l). Therefore, KIF4A was upregulated in BC and affected the prognosis of BC patients, which may be the indispensable target of miR‐379‐5p.

### MiR‐379‐5p inhibits proliferation, migration, and invasion of BC cells

**FIGURE 3:**
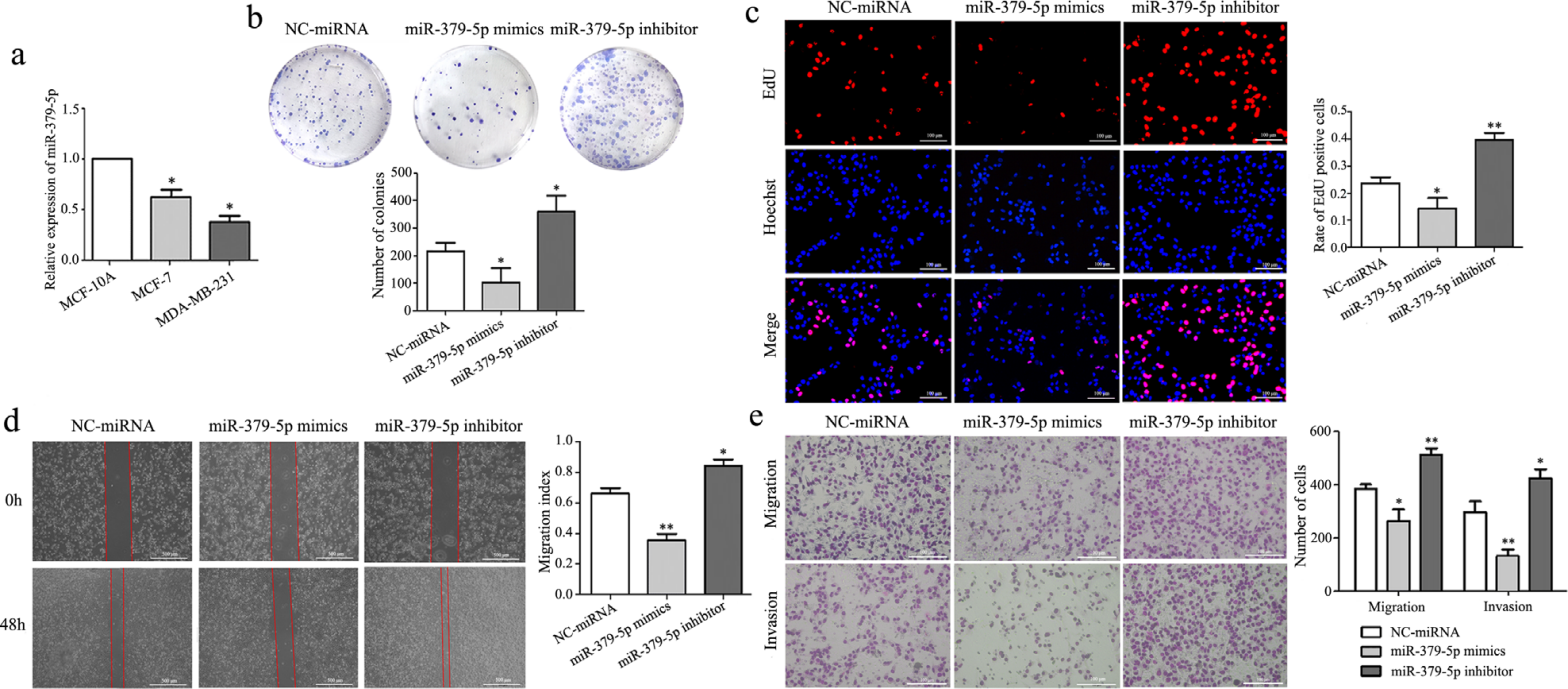
MiR‐379‐5p inhibits proliferation, migration, and invasion of BC cells. (a) QRT‐PCR shows the relative expression of miR‐379‐5p in MCF‐10A, MCF‐7, and MDA‐MB‐231 cells. (b) The colony formation assay was performed in MDA‐MB‐231 cells after transfection. (c) The proliferation ability of transfected MDA‐MB‐231 cells was analyzed by EdU assay. Scale bars, 100 μm. (d) Wound healing assay of MDA‐MB‐231 cells after transfection at 0 and 48 h. Scale bars, 500 μm. (e) A Transwell assay was performed to analyze the invasion and migration abilities of MDA‐MB‐231 cells after transfection. Scale bars, 100 μm. All data were presented as mean ± SD. *p < 0.05; **p < 0.01)

QRT‐PCR results showed that the expression of miR‐379‐5p in the BC cells (MDA‐MB‐231 and MCF‐7) was significantly reduced compared with human normal mammary epithelial cells (MCF‐10A). Meanwhile, the expression of miR‐379‐5p in MDA‐MB‐231 cells was lower than that of low‐invasive MCF‐7 cells (Figure 3a). Therefore, we chose MDA‐MB‐231 cells as the essential tool for this study. To investigate the potential role of miR‐379‐5p in BC, we overexpressed or knocked down the expression of miR‐379‐5p in MDA‐MB‐231 cells. Compared with the negative control, the number of cell colonies with miR‐379‐5p overexpressed was significantly reduced, while the number of cell colonies with miR‐379‐5p downregulated was significantly increased (Figure 3b). The EdU assay showed that upregulation of miR‐379‐5p significantly inhibited the proliferation of MDA‐MB‐231 cells compared with the negative control. In contrast, downregulation of miR‐379‐5p significantly promoted the proliferation of MDA‐MB‐231 cells (Figure 3c). The wound‐healing assay revealed that overexpression of miR‐379‐5p significantly inhibited the migratory ability of MDA‐MB‐231 cells compared with the negative control. And the down‐regulation of miR‐379‐5p significantly promoted the migratory ability (Figure 3d). Transwell assay showed that the overexpression of miR‐379‐5p significantly inhibited the migration and invasion of MDA‐MB‐231 cells. Similarly, the downregulation of miR‐379‐5p promoted MDA‐MB‐231 cells migration and invasion (Figure 3e). Therefore, miR‐379‐5p can be used as the potential marker of BC and significantly inhibit BC's proliferation, migration, and invasion.

### KIF4A is a direct target of miR‐379‐5p and can reverse the inhibitory effect of miR‐379‐5p on BC cells

**FIGURE 4:**
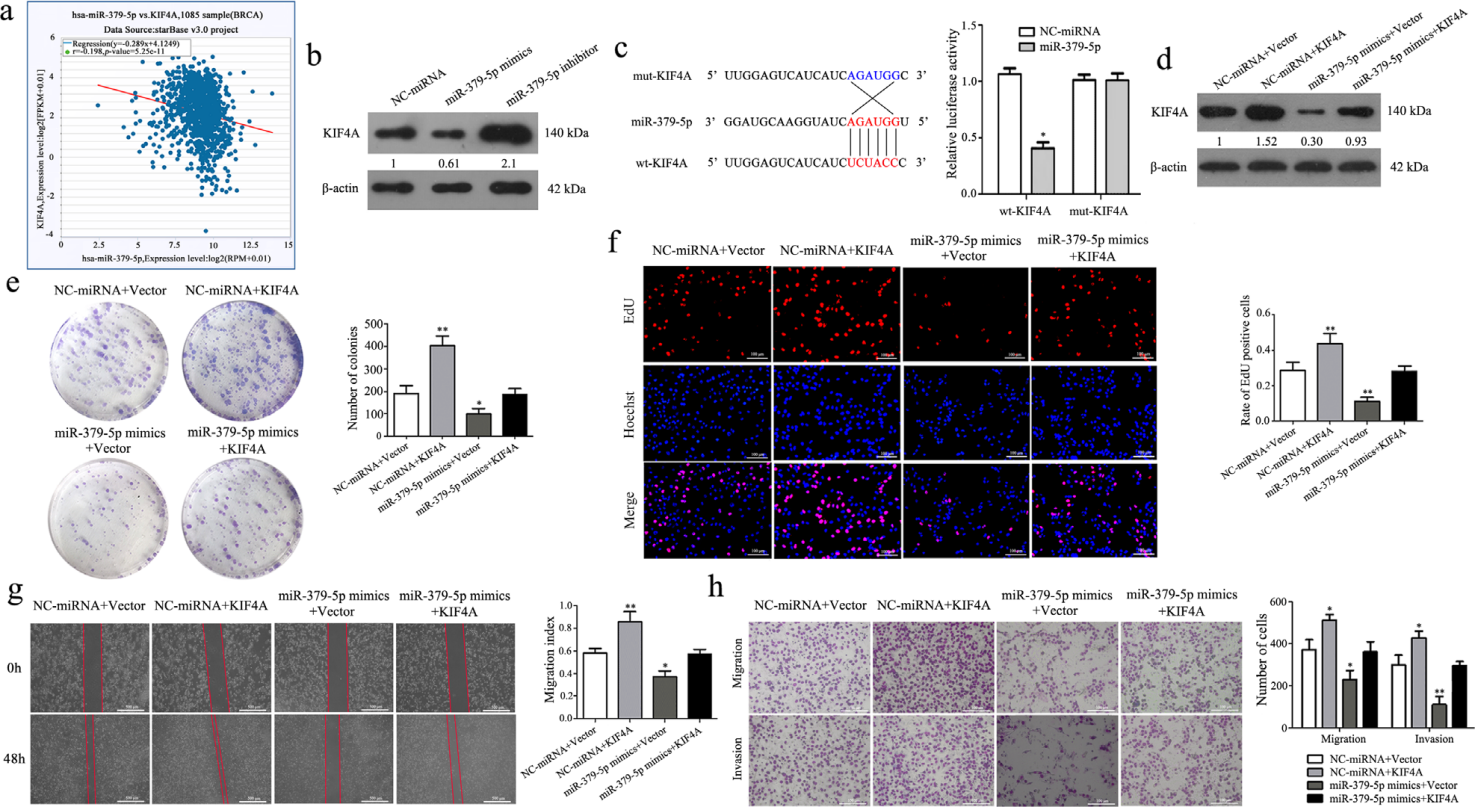
KIF4A is the direct target of miR‐379‐5p and can reverse the inhibitory effect of miR‐379‐5p on BC cells. (a) Correlation curve between the expression of miR‐379‐5p and KIF4A in BC (*r* = −0.198, *p* < 0.05). (b) The expression of KIF4A in MDA‐MB‐231 cells after transfection. (c) Luciferase reporter assays were used to confirm miR‐379‐5p directly targeting the 3'UTR of KIF4A in HEK‐293 T cells. (compared to NC‐miRNA. Three experiments were done). (d) The expression of KIF4A protein was measured by western blot in different cells. (e) The colony formation assay was performed in MDA‐MB‐231 cells after transfection. (f) The EdU assay analyzed the proliferation ability of MDA‐MB‐231 cells after transfection. Scale bars, 100 μm. (g) Migration ability was tested by wound healing assay after transfection. Scale bars, 500 μm. (h) Migration and invasion of MDA‐MB‐231 were examined by transwell assay after transfection. Scale bars, 100 μm. (All data were presented as mean ± SD. *p < 0.05; **p < 0.01)

To explore the link between KIF4A and miR‐379‐5p, we performed correlation analysis with the starBase database. The results confirmed that the expression level of miR‐379‐5p was negatively correlated with KIF4A, and the expression of KIF4A decreased with the increase of miR‐379‐5p expression in BC (Figure 4a). Western blot also showed that MDA‐MB‐231, which was overexpressed by miR‐379‐5p mimics, inhibited the expression of KIF4A protein (Figure 4b). To prove if the predictive sequences binding to miR‐379‐5p on the 3'‐UTR of KIF4A was responsible for this regulation, we transfected wt KIF4A or mut KIF4A plasmid and miR‐379‐5p plasmid or NC‐miRNA into HEK‐293 T cells. The results showed that the luciferase activity in HEK‐293 T cell line cotransfected with miR‐379 and wt‐KIF4A was lower than that in HEK‐293 T cell line cotransfected with NC‐miRNA and wt‐KIF4A. But the luciferase activity did not change significantly in HEK‐293 T cell line cotransfected with miR‐379 and mut‐KIF4A and cotransfected with NC‐miRNA and mut‐KIF4A (Figure 4c). The above results indicated that KIF4A is the direct target of miR‐379‐5p in BC.

To determine whether miR‐379‐5p affects the biological function of BC cells by targeting KIF4A, we overexpressed KIF4A together with miR‐379‐5p mimics in MDA‐MB‐231 cells. Western blot results showed that compared with the NC‐miRNA+Vector group, the expression of KIF4A was upregulated in the NC‐miRNA+KIF4A group and downregulated in the miR‐379‐5p mimics + vector group. However, this effect of miR‐379‐5p mimics + Vector group on the occurrence of breast cancer was partly abolished after cotransfection with KIF4A (Figure 4d). Colony formation and EdU assays showed that the overexpression of KIF4A significantly promoted the proliferation ability compared with the negative control. In addition, we found that miR‐379‐5p mimics inhibited the proliferation ability of MDA‐MB‐231 cells, and the overexpression of KIF4A can reverse the inhibitory effect of miR‐379‐5p on the proliferation of MDA‐MB‐231 cells (Figure 4e and f). Wound‐healing assays revealed that overexpression of KIF4A reversed the inhibitory effect of miR‐379‐5p on cell migration (Figure 4g). Similarly, transwell assay showed that KIF4A overexpression promoted the migration and invasion of MDA‐MB‐231 cells and reversed the inhibitory effect of miR‐379‐5p on the migration and invasion of MDA‐MB‐231 cells (Figure 4h). Therefore, miR‐379‐5p inhibited the proliferation, migration, and invasion of BC by targeting KIF4A.

## DISCUSSION

With the continuous innovation of biotechnology and the rapid development of novel high‐throughput technologies, microarray‐based expression profiling studies have confirmed that different grades of gene expression differences in breast cancer may have prognostic value.^26^, ^27^ Studies have shown that miRNAs are differentially expressed in different BC subtypes, providing critical value for diagnosis of the disease, treatment, and prognosis.^28^ In this study, the expression of miR‐379‐5p was significantly lower in BC tissues than in normal breast tissues. Previous studies have also confirmed that miR‐379 is the effective tumor suppressor in BC, mediated partly through the regulation of COX‐2.^29^ Through qRT‐PCR, the expression of miR‐379‐5p in the highly aggressive MDA‐MB‐231 cells was considerably lower than that of MCF‐7 and MCF‐10A cells. Therefore, this further validates the results of bioinformatic analysis. It has demonstrated that smoking and betel quid chewing can inhibit the expression of miR‐379 and upregulate DNMT3B, thus promoting the methylation of ADHFE1 and ALDH1A and promoting the oncogenic activity.^30^ The EdU and colony formation assays proved that overexpression of miR‐379‐5p could inhibit BC cell proliferation. Through wound healing and transwell assays, overexpression of miR‐379‐5p significantly inhibited the migration and invasion of BC cells. Overexpression of miR‐379‐5p inhibits cell proliferation and promotes apoptosis in various tumor contexts in vitro, and prolongs survival in mice.^31^ In summary, miR‐379‐5p is closely related to the development of breast cancer and may have important clinical significance for the diagnosis, treatment and prognosis of tumors.

We used multiple online databases to predict the target genes of miR‐379‐5p. After obtaining the intersection, we constructed the PPI network for the 89 target genes to study the relationship between the target proteins and identified ten hub genes. The proteins encoded by these genes are vital nodes in the PPI network. Target genes are mainly enriched in cell–cell adhesion, cell division, mismatch repair, protein binding, etc. We found that only KIF4A expression was strongly associated with patient prognosis and that KIF4A expression was higher in cancer tissues than in normal tissues. KIF4A has involved chromosome condensation and cytokinesis during mitosis. It may affect the spindle morphology and chromosome arrangement of mouse oocytes by regulating tubulin acetylation and promote cell proliferation by regulating the p21 promoter but not affecting cell apoptosis.^32^

Western blot showed that when the expression of miR‐379‐5p was upregulated, the expression of KIF4A was lower. Next, we used the dual‐luciferase reporter assay system to confirm that KIF4A was the target gene of miR‐379‐5p. It has been stated that the increased expression of KIF4A can affect the degree of immune cell infiltration, and it may be involved in the regulation of immune tolerance of cholangiocarcinoma cells.^33^ KIF4A is a direct downstream target of FOXM1c and is overexpressed in HCC tissues.^34^ Meanwhile, it has been confirmed that the expression of KIF4A is positively correlated with malignant features of BC, which may be the target gene of miR‐335.^35^ However, few studies have demonstrated the effect of KIF4A on the function of BC cells. Therefore, after the plasmid successfully transfected MDA‐MB‐231 cells, we performed a series of experiments to demonstrate a targeting relationship between miR‐379‐5p and KIF4A. We found that KIF4A can reverse the inhibitory effect of miR‐379‐5p on BC cell migration, invasion, and proliferation. Similar studies have shown that exosomal miR‐195 inhibited osteosarcoma cell proliferation and antiapoptosis in vitro, inhibited tumor growth in vivo, and reduced tolerance to chemotherapeutic drugs by targeting KIF4A.^36^ These results suggest that miR‐379‐5p inhibits BC progression by downregulating KIF4A.

In summary, our data indicate that miR‐379‐5p is downregulated in BC and inhibits BC proliferation, migration, and invasion. MiR‐379‐5p serves as an important molecular marker with the potential to be a novel promising candidate for the prognosis and therapy of BC. As the target gene of miR‐379‐5p, KIF4A is overexpressed in BC and is significantly related to patient prognosis. Therefore, it was verified that miR‐379‐5p inhibits the proliferation, migration and invasion of BC by targeting KIF4A.

## CONFLICT OF INTEREST

No authors report any conflict of interest.

## Supporting information


**Figure S1** The survival analysis based on the expression of ESPL1, IQGAP3, RNASEH2A, AURKA, PRR11, PRC1, GAS2L3, CDCA8, and CEP55 in BC patients.
