# Supplementary figures and images for: MiR‐379‐5p inhibits the proliferation, migration, and invasion of breast cancer by targeting KIF4A
Source: Thorac Cancer. 2022 May 24;13(13):1916–24. doi: 10.1111/1759-7714.14437 (PMC9250835; doi:10.1111/1759-7714.14437)

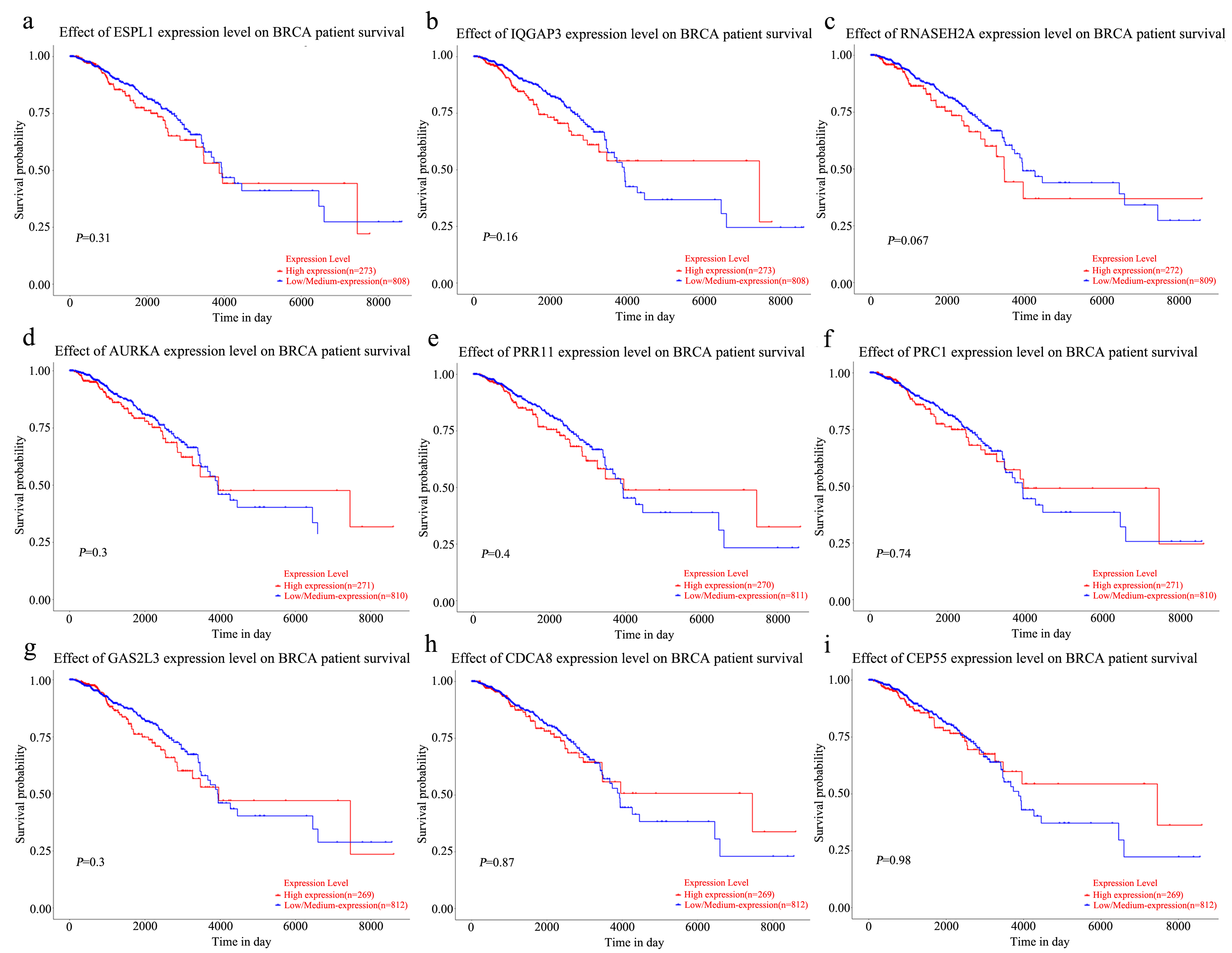

Supplement: Supplementary file 1 — Figure S1 The survival analysis based on the expression of ESPL1, IQGAP3, RNASEH2A, AURKA, PRR11, PRC1, GAS2L3, CDCA8, and CEP55 in BC patients. [file TCA-13-1916-s001.tif]
